# Multiple Roles of Peripheral Immune System in Modulating Ischemia/Hypoxia-Induced Neuroinflammation

**DOI:** 10.3389/fmolb.2021.752465

**Published:** 2021-11-22

**Authors:** Liang Guo, Lingling Zhu

**Affiliations:** ^1^ Beijing Institute of Basic Medical Sciences, Beijing, China; ^2^ University of Nanhua, Hengyang, China; ^3^ Anhui Medical University, Hefei, China

**Keywords:** peripheral inflammation, neuroinflammation, brain-peripheral crosstalk, hypobaric hypoxia, ischemic hypoxia

## Abstract

Given combined efforts of neuroscience and immunology, increasing evidence has revealed the critical roles of the immune system in regulating homeostasis and disorders of the central nervous system (CNS). Microglia have long been considered as the only immune cell type in parenchyma, while at the interface between CNS and the peripheral (meninges, choroid plexus, and perivascular space), embryonically originated border-associated macrophages (BAMs) and multiple surveilling leukocytes capable of migrating into and out of the brain have been identified to function in the healthy brain. Hypoxia-induced neuroinflammation is the key pathological procedure that can be detected in healthy people at high altitude or in various neurodegenerative diseases, during which a very thin line between a beneficial response of the peripheral immune system in maintaining brain homeostasis and a pathological role in exacerbating neuroinflammation has been revealed. Here, we are going to focus on the role of the peripheral immune system and its crosstalk with CNS in the healthy brain and especially in hypobaric or ischemic hypoxia-associated neuroinflammation.

## Introduction

The proper function of central nervous system (CNS) is protected by the immune system, including the CNS-resident and the peripheral immunity. The cerebral immune microenvironment is well-controlled in the healthy brain by the interfaces between blood, cerebrospinal fluid (CSF), and brain. The endothelial or epithelial interface structures are generally referred to as “barriers,” with tight junctions between adjacent cells to separate the brain from circulating molecules and effector immune cells. Thus far, several distinct barriers have been described: (1) blood-brain barrier (BBB) with luminal tight junctions between the endothelial cells of cerebral blood vessels, (2) arachnoid blood-CSF barrier (BCSFB), also called meningeal barrier, consisting of tight junctions between arachnoid cells, (3) choroid plexus (CP) BCSFB with apical tight junctions between epithelial cells in CP, and (4) ventricular barrier with tight junction formed by adjacent ependymal cells to separate parenchyma from CSF (Ek et al., 2012; Schmitt et al., 2017). Under homeostatic conditions, BBB is considered as absolute immunological barriers that block leukocytes entry into the parenchyma, while BCSFB comprised of fenestrated vasculature enveloped by tightly regulated endothelium are believed to serve as active and selective immune-skewing gates (Shechter et al., 2013a; Demeestere et al., 2015). Therefore, the term “barrier” is somewhat misleading for highlighting the selective permeability of the morphological barriers. To better interpret their roles as entry sites for peripheral leukocytes, the barrier structures have also been reviewed as “blood-brain interfaces”: (1) the BBB enclosing the microvessels and glial limitans within parenchyma; (2) the blood-meningeal interfaces between pia-arachnoid blood vessels and CSF of subarachnoid space, pial blood vessels of pial basement membrane and glial limitans, and across dural blood vessels; and (3) the CP interfaces between fenestrated capillaries and CP epithelial cells, and the apical choroidal region and cerebral ventricular CSF (Spadoni et al., 2017; Benakis et al., 2018). Whatever the terminology, these barriers or interface structures between blood, brain, and CSF are critical to determine the trafficking of circulating leukocytes into the brain under both physiological and pathological conditions.

**FIGURE 1:**
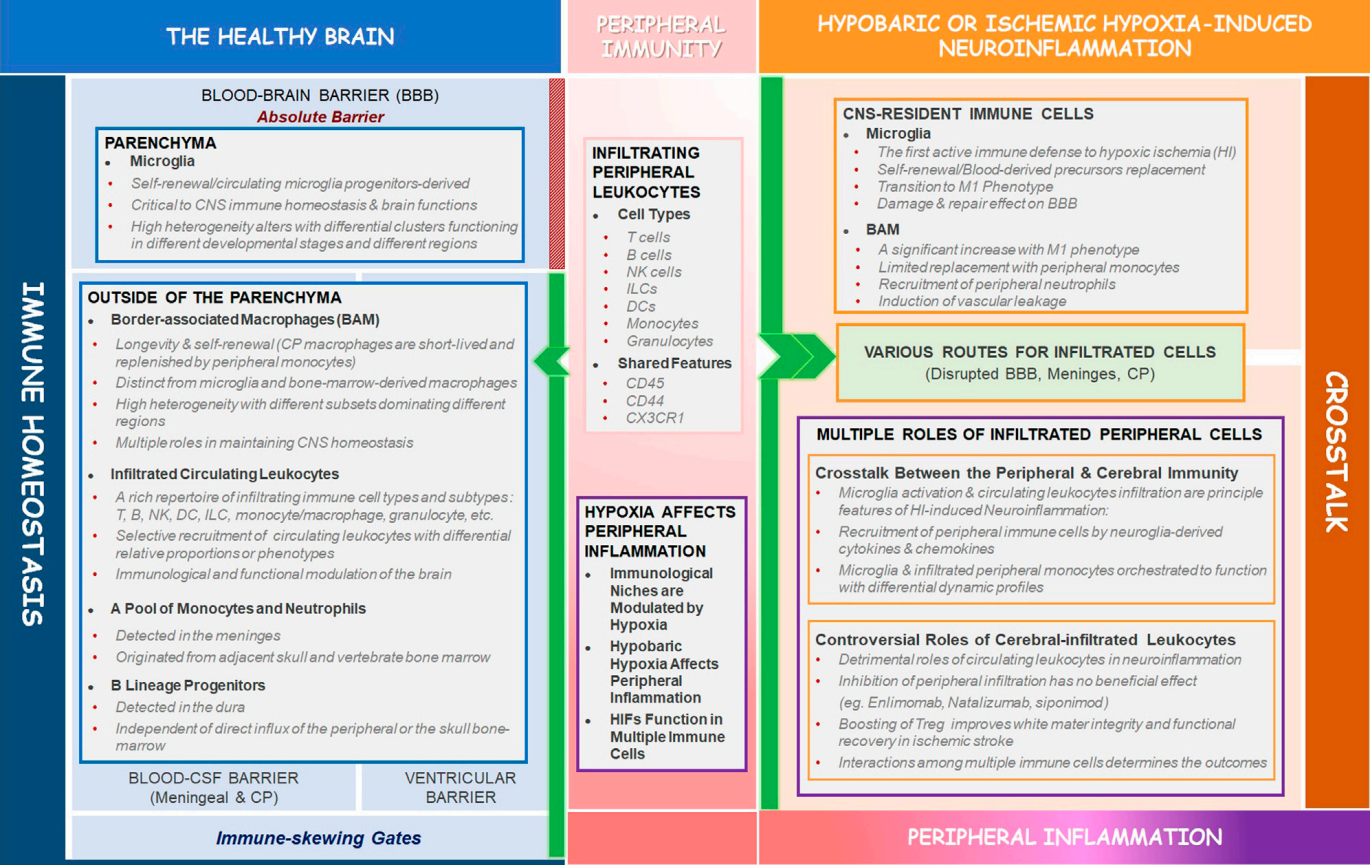
Schematic summary of the review. In the heathy brain, BBB acts as an absolute barrier to separate the parenchyma from circulating leukocytes, while outside of the parenchyma, the BCSFBs serve as active and selective immune-skewing gates to recruit circulating leukocytes with some shared markers (CD45, CD44, CX3CR1) to the interfaces (meninges, CP, and perivascular space) between the CNS and peripheral immunity (the left blue box). Hypoxia can play critical roles in affecting peripheral inflammation under both physiological and pathological conditions (the middle pink box). In hypobaric- or ischemic hypoxia-induced neuroinflammation, the circulating leukocytes infiltrate into the parenchyma through disrupted BBB and alternative routes (meninges, CP). The CNS-residential immune cells (microglia and BAM) and infiltrated leukocytes interact with each other to determine the outcomes of brain injury. Controversial roles of the infiltrated peripheral immune cells in regulating hypoxia-induced neuroinflammation are of great importance (the right orange box) and are the hotspot of the future study.

Hypoxia, which can be detected in healthy people at high altitude or in various pathological conditions as trauma, stroke, inflammation, autoimmunity, and neurodegenerative diseases, induces severe brain damage, leading to deficits of cognition, study, or memory (Chen et al., 2020). Neuroinflammation is a hallmark of essentially all CNS pathologies (Lyman et al., 2014; Mckenzie et al., 2017). Hypoxia-induced neuroinflammation is contributed by both CNS-residential and infiltrating peripheral immune cells. A bidirectional crosstalk between the peripheral and CNS immunity in response to hypoxia has been revealed and multiple cell types have been suggested to exert differential roles in the generation and progression of hypoxic brain injuries (Li et al., 2017). Here, we will review the heterogeneity of the brain in healthy conditions and under hypobaric or ischemic hypoxia, discuss the roles of peripheral inflammatory cells in hypoxic brain injuries, including the crosstalk between the peripheral and CNS-residential immunity and multiple roles of infiltrating populations in regulating hypoxic brain damage (Figure 1).

## The Central Nervous System Immunity in the Healthy Brain

The CNS is a compartmentalized organ composed of the parenchyma enveloped by the meningeal layers, the ventricles containing CP and CSF, and the brain barriers (Shechter et al., 2013b). The CNS was long considered as an “immune privileged” site due to the lack of obvious lymphatic vessels and the BBB restricting the infiltration of peripheral immune cells and molecules. However, functional lymphatic vessels have been “rediscovered” recently in the meninges, assisting in draining CNS/CSF-derived soluble molecules. Notably, classical immune cells such as T cells, B cells, and CX3CR1^+^ myeloid cells were detected in the CNS lymphatic vessels in the healthy brain (Louveau et al., 2015). Moreover, in contrast to the BBB, the semi-permeable BCSFB, comprising fenestrated endothelium, serves as active and selective immune-skewing gate in the healthy brain (Shechter et al., 2013a). Therefore, it is now accepted that in the healthy brain, the immune privilege level varies dramatically between CNS compartments, with the highest privilege in the parenchyma and more communications with the peripheral immunity outside of the parenchyma.

### Microglia

The main inflammatory cells within the CNS are microglia, which are seeded into the brain during embryogenesis and are the only resident leukocytes within the parenchyma where they function in neuronal synapse sculpting and immune surveillance in the steady-state (Prinz and Priller, 2014; Colonna and Butovsky, 2017). However, the origin of the accumulated microglia has long been disputed, even utilizing the same animal model that subjects mice to irradiation and transplantation. Several lines of evidence supported that the circulating microglial progenitors that were originated from hematopoietic stem cells were capable of infiltrating into the CNS and contributed to the microglia pool (Hess et al., 2004). While other studies suggested that the maintenance and local expansion of microglia were solely dependent on the self-renewal of CNS-resident cells under both physiological and pathological conditions (Massengale et al., 2005; Solomon et al., 2006; Ajami et al., 2007). Of note, it has been argued that the infiltration of circulating microglia progenitors into the brain might be attributed to the irradiation-induced disruption of the BBB and the transplantation of circulating progenitors acquired by mechanically flushing that would not enter the bloodstream under physiological conditions (Ajami et al., 2007). Recent study using chimeric animal model obtained by parabiosis that requires neither irradiation nor transplantation is also in favor of the notion that microglia are capable of self-renewal, functioning independently of bone marrow-derived progenitors throughout adult life (Ajami et al., 2007).

In addition to the critical functions of microglia in immune surveillance, our understanding has also been evolved with the respect of their roles in developmental and physiological plasticity (Schafer and Stevens, 2015; Stevens and Schafer, 2018). A young individual born deprived of microglia because of homozygous mutation in CSF1R, underwent severe congenital brain anomalies (Oosterhof et al., 2019). Furthermore, it is increasingly recognized that heterogenous microglia may underlie the diverse roles they play in different developmental and pathological conditions. Single-cell sequencing (scRNA-seq) technology makes it possible to manifest details of the microglial heterogeneity. Nine clusters, characterized by a differentially expressed gene set, have been identified to be distinct clusters of human microglia in the healthy brain, with different subsets involved in homeostasis, proliferation, interferon response, and antigen presentation. Notably, some clusters were enriched for disease-related genes (Olah et al., 2020). Consistent with its peripheral counterpart macrophage, microglia could also rapidly change states in response to stimuli from the environment. Evidence has indicated that the heterogeneity of microglia altered during different development stages and in different regions. In mice, nine unique states of microglia were revealed across all stages. Aging triggered a shift toward a more immunogenic profile rather than inducing the appearance or disappearance of any cluster. The diversity of microglia was found to peak at the youngest ages (E14.5 and P5) and decreased over time. Several states, including chemokine-enriched inflammatory microglia, persisted throughout the lifespan or increased in the aged brain (Hammond et al., 2019). Single-cell RNA sequencing of microglia sorted from various regions of mouse brain revealed seven clusters of microglia across developmental stages. In adult mice, most microglia expressed similar homeostatic genes and had only limited transcriptomic heterogeneity across brain regions. However, the diversity of the early postnatal brain was much higher and a proliferative region-associated microglia subtype which shared a transcriptomic signature with degenerative disease-associated microglia was found mainly in developing corpus callosum and cerebellar white mater (Li et al., 2019).

### Border-Associated Macrophages

Outside the parenchyma, embryonically originated border-associated macrophages (BAMs) and multiple surveilling leukocytes capable of migrating into and out of the brain play critical roles in maintaining CNS homeostasis. It is evident that, under physiological circumstances, recruitment of leukocytes from peripheral immune system is restricted and tightly regulated at the interface (the meninges, CP, and perivascular space) between CNS and peripheral immunity. In undiseased brain, the neuroimmune interaction was suggested to be active around the dural sinuses where the peripheral adaptive immunity surveyed the CNS-derived antigens in the CSF (Rustenhoven et al., 2021). Interestingly, the meninges not only host a rich repertoire of circulating immune cells, but also contain a pool of monocytes and neutrophils originated from adjacent skull and vertebrate bone marrow rather than the blood, which further increases the heterogeneity outside of the parenchyma (Cugurra et al., 2021). The conventional flow cytometry method of 14–16 parameter staining panel is limited to mapping leukocytes diversity across the entire CNS in the healthy brain and particularly in pathology when the influx of peripheral cells increases the difficulty to unambiguously distinguish between reactive leukocyte populations. With the advent of scRNA-seq and mass cytometry technology, a substantial amount of complexity of the leukocytes within the CNS under physiological and pathological conditions in animal models has been revealed.

Border-associated macrophages (BAMs) are macrophages residing in the meninges, CP, and perivascular regions, which originate prenatally from the same pool of yolk sac hematopoietic progenitors and migrate to the brain at the same developmental time as microglia (Goldmann et al., 2016). The divergence between microglia and BAM occurred when microglia entered the parenchyma to achieve unique signatures. It has been reported that at E14.5, a *Ms4a*7^+^ cluster of microglia in mouse brain, expressing transcripts of both macrophage and mature microglia marker genes, also possessed a transcriptional overlap with BAM, which suggested this subpopulation might be an intermediate to give rise to perivascular macrophages or mature microglia (Hammond et al., 2019). Moreover, different clusters of microglia and choroid plexus BAM showed close juxtaposition on tSNE plot, also implicating the overall similarity between BAM and microglia (Li et al., 2019). Several transcriptional signatures have been revealed to distinguish BAM from microglia and other myeloid cells. In murine brain, almost 80% of total brain immune cells are CD45^low^CX3CR1^+^CD11b^+^ myeloid, among which BAM that lack typical microglial Siglec-H expression and instead express CD206 and CD38 encompass the meningeal, perivascular, and CP regions (Galea et al., 2005; Mrdjen et al., 2018). In rat, monkey, and human brains, the scavenger receptor CD163, which is involved in response to inflammation (Etzerodt and Moestrup, 2013), has been suggested as a typical marker for BAM by several groups (Fabriek et al., 2005; Kim et al., 2006; Zhang et al., 2011). However, CD163 is expressed on multiple peripheral immune subsets, especially on M2 phenotype of macrophages, which makes it ambiguous to identify BAM by CD163 alone and thus more unique antigens are required to BAM identification instead of or in combination with CD163.

Though exhibiting similar phenotype, BAMs are a distinct population from microglia and bone-marrow-derived macrophages. Recent single-cell RNA analysis demonstrated a specific BAM core gene signature (*Apoe*, *Ms4a7*, *Ms4a6c*, *Clec4a1*) and heterogeneity within BAMs under physiological conditions (Van Hove et al., 2019). Four subsets of BAMs have been distinguished by differential expression of CD38 or Lyve1, MHCII and CCR2, among which CCR2^+^ subset may represent a fresh hematopoietic myeloid cell to replace BAMs accumulating in the CP. The population that was positive for CD38 and Lyve1 made up 30% of BAM (Mrdjen et al., 2018). Furthermore, some subsets of BAM were found to localize exclusively in specialized anatomical locations: for example, the MHC II^−^/Lyve1^+^ subpopulation was mainly present in the dura mater where the BAMs positive for both MHC II and Lyve1 were absent (Mrdjen et al., 2018).

BAM have been proven to form stable populations as a result of their longevity and their capacity for self-renewal, except for CP macrophages that have a shorter life span and are replenished by peripheral monocytes (Gomez Perdiguero et al., 2015; Sheng et al., 2015; Goldmann et al., 2016). Border macrophages participate in CNS homeostasis through multiple ways, including affecting vascular permeability and BBB integrity, carrying out scavenger functions, filtering and draining the CNS, scanning for invading pathogens, and presenting antigens to circulating lymphocytes (Fabriek et al., 2005; Coles et al., 2017; Kierdorf et al., 2019). BAMs were also implied in the regulation of metabolic processes. In mice that were fed with a high-fat diet, glucose uptake into the CNS was decreased because of reduction of glucose transporter I (GLUT I) in CNS vascular endothelial cells (EC), in response to which perivascular macrophages secreted vascular endothelial growth factor (VEGF) to enhance GLUT I expression in ECs and thus restored glucose uptake in the CNS (Jais et al., 2016). Moreover, *slc40a1*, a gene that encodes ferroportin, has been identified as a specific marker for perivascular macrophages (Zeisel et al., 2015), implying a potential role of BAMs in regulating iron metabolism. A growing number of studies have been focusing on the functions of BAM in undiseased brain, however, there is still a long way to go toward uncovering the full spectrum of their physiological roles.

### Physiological Traffic of Peripheral Immune Cells

In spite of great heterogeneity of infiltrating cells, some features are shared by most of the immune populations in the brain: CD45 and CD44 are suggested as markers for infiltrating cells whose expression of CX3CR1 are usually elevated to relate to their migration to the brain (Mrdjen et al., 2018). Single cell analysis identified an abundant and complex immune cell landscape among infiltrating (CD45 high) cells, including dendritic cells (DCs), innate lymphoid cells (ILCs), T cells, B cells, monocytes, and even granulocytes (neutrophils, eosinophils, mast cells, etc.) (Goldmann et al., 2016; Mrdjen et al., 2018), with differential relative proportions when compared to their peripheral counterparts and thus indicated a selective interaction between the brain and the blood (Korin et al., 2017).

CD45^hi^CD11c^hi^MHC II^hi^ DC cells, derived from migrating pre-DC progenitors of bone marrow, have been detected localized to the meninges and CP with an intrinsic requirement of Flt3 ligand (Anandasabapathy et al., 2011). Single-cell analysis has identified three subsets of DCs in the distinct border regions of the steady-state CNS by the expression of CD11b and CD24: conventional (c) DC1s predominantly located in the CP, cDC2s represented in the meninges and perivascular spaces (the latter of which is populated by no or only very low numbers of DCs), and plasmacytoid (p) DCs restricted to the dura (Mrdjen et al., 2018; Mundt et al., 2019; Van Hove et al., 2019). CSF drainage via both nasal and meningeal lymphatic vessels to cervical lymph node (CLN) is suggested to allow antigen-presenting cells (APCs), especially DCs, to continuously sample and present neuro-antigens to the periphery, which might contribute to induce peripheral tolerance in the healthy brain and initiate CNS-specific adaptive T cell immunity on exposure to pathogens (Harris et al., 2014; Louveau et al., 2018). Though DCs in the CNS are equipped with antigen-presenting machinery to stimulate T cells, activated T cells have no access to the CNS under cerebral steady-state (Anandasabapathy et al., 2011; Harris et al., 2014).

Most of the T cells within the CSF/meninges are memory T cells in both human and mouse (Zheng et al., 2004; Ransohoff and Engelhardt, 2012). P-selectin and intracellular adhesion molecular 1 (ICAM-1) were abundantly expressed in the CP and meninges, recruiting CD4^+^/CD45RA^−^/CD27^+^/CD69^+^-activated central memory T cells that expressed high levels of CCR7, L-selectin, and P-selectin glycoprotein ligand 1 (PSGL-1) through interactions of P-selectin/P-selectin ligands and ICAM-1/lymphocyte function-associated antigen 1 (LFA-1) (Kivisakk et al., 2003). Besides patrolling the brain, CNS-specific T cells also function in neurogenesis and spatial learning and memory in mouse model by releasing neurotrophic factors and cytokines (interferon(IFN)-γ and interleukin(IL)-4) or by interacting with microglia (Butovsky et al., 2006; Ziv et al., 2006; Derecki et al., 2010; Filiano et al., 2016). Adoptive transfer of brain antigenreactive CD4^+^ T cells was sufficient to partially rescue the impaired learning behavior of OTII mice (Radjavi et al., 2014). B cells are abundant in mouse meninges, functioning in early oligodendrogenesis and myelination (Tanabe and Yamashita, 2018). In contrast to T cells, the contribution of B cells to learning behavior is minimal as evidenced by no Morris water maze (MWM) impairment in B-cell-deficient mice (Radjavi et al., 2014). Very recently, murine dura B cells have been reported to exhibit slow turnover and long-term residency in the meninges. Of note, B lineage progenitors at the pro-B cell stage typically not identified outside of bone-marrow were also detected in the non-diseased dura, independent of direct influx from the peripheral or the skull bone-marrow (Schafflick et al., 2021). This study provided an alternative source of immune cells outside of the parenchyma other than the circulating leukocytes, indicating an active role of meninges in the immuno-modulation of the brain.

Not only T and B lymphocytes, but also innate lymphoid cells (ILCs), especially ILC2, patrol the meninges and CP of the healthy CNS. It has been revealed that CNS-associated ILCs exert either a pathological or neuroprotective role in the context of spinal cord injury, their role in CNS homeostasis is not understood yet. A higher proportion of CD62L^+^ NK cells in the CNS than in the peripheral blood was also identified, which are associated with a relatively mature phenotype and a stronger cytotoxic function (Peng et al., 2013). These data demonstrate a rich repertoire of immune cells with high diversity in the meninges. These findings highlight the complexity of the cerebral immune microenvironment and the selectivity of the brain for specific immune populations. Furthermore, the functional relevance of the unique features characterizing the infiltrating cells are in urgent need to be extensively explored to unravel the surveillance mechanisms dominated by cerebral immune cells.

## Crosstalk Between the Peripheral and Central Nervous System-Resident Immunity in Response to Ischemia/Hypoxia

### Hypoxia-Induced Polarization of Microglia and Border-Associated Macrophage and Their Effects on Blood-Brain Barrier Integrity

Hypoxia in the brain has been observed in healthy people ascending to high altitude or in various pathological conditions (Supplementary Table S1; Supplementary Reference List). As the major resident immune cells in the CNS, microglia act as the first active immune defense to multiple stimuli. Microgliosis, which is characterized by an increase in the number of microglia, is a hallmark of essentially all CNS pathologies. Active microglia precursor recruitment from the bloodstream across the BBB has been reported in a variety of diseases. However, in amyotrophic lateral sclerosis (ALS) model whose pathogenesis is intimately related to brain ischemic hypoxia, the large increase in microglia was attributed to the self-renewal capacity of CNS-resident microglia (Ajami et al., 2007). It has been reported that hypoxia-inducible factor 1α (HIF-1α) is critical to regulate microglia activation in ischemia/hypoxia conditions, inducing neuron degeneration. HIF-1α knockdown in microglia abrogated hypoxia-induced phagocytosis, production of intracellular reactive oxygen species (ROS) and tumor-necrosis factor α (TNF-α), which protected neuronal survival in the acute phase of ischemic stroke in mice model (Bok et al., 2017). Hypoxia has also been indicated in microglia autophagy, contributing to neural inflammation injury in the murine focal cerebral ischemic model (Yang et al., 2015).

In response to hypoxia in the CNS, microglia as the resident macrophage in the brain would also transdifferentiate between proinflammatory M1 and immunosuppressive M2 phenotype. Microglia toward M1 phenotype is one of the driving forces of neurodegenerative disorders by impairing cellular functions and inducing growing neuronal damage via the production of ROS, reactive nitrogen species, and a variety of inflammatory cytokines. Emerging evidence has manifested the role of hypoxia in the transition of microglia to M1 phenotype (Habib et al., 2013; Zhang et al., 2017; Butturini et al., 2019). Increased expression of M1 markers (CD86, tumor necrosis factor α (TNF-α), IL-6), chemokine C-C motif ligand 2 (CCL2) and CCL3) and decreased level of M2 markers (Arginase-1, CD206, IL-4 and IL-10) in microglia were caused by acute hypoxia through Toll-like receptor 4 (TLR4)-activation of nuclear factor-kappa B (NF-ĸB) (Zhang et al., 2017). Hypoxia also generated oxidative stress and thus activated M1 microglia by signal transducer and activator of transcription 1 (STAT1) phosphorylation and S-glutathionylation in BV2 cells (Butturini et al., 2019). Moreover, sex steroid hormone (estrogen and progesterone) was revealed to dampen hypoxia-induced neuroinflammation by switching microglia from M1 to immunosuppressive M2 phenotype in both BV-2 cell line and primary rat cerebral cortex-derived microglia (Habib et al., 2013; Habib et al., 2014).

The roles of microglia in regulating BBB integrity were controversial. The pathogenic role for activated microglia in BBB disruption has been implied in ischemic stroke and experimental autoimmune encephalomyelitis/multiple sclerosis (EAE/MS) mouse models that are both closely related to severe hypoxia (Adams et al., 2007; Jolivel et al., 2015). However, other groups reported that under chronic mild hypoxia conditions, microglia were recruited and activated around damaged vessels, capable of repairing the leaky blood vessels and maintaining vascular integrity in brain and spinal cord via fibrinogen-Mac-1 interaction. While depletion of microglia led to increased astrocyte-vascular uncoupling, tight junction proteins loss, and BBB leaky (Halder and Milner, 2019; Halder and Milner, 2020).

BAMs also play active roles in response to cerebral hypoxia. In stroke, hypoxia is a common occurrence and associated with poor clinical and functional outcomes. CD163, a scavenger receptor functioning in inflammatory response and connective tissue remodeling (Kowal-Bielecka et al., 2013), is identified to expressed in multiple peripheral immune populations as M2 macrophages and DC subset and has been suggested to be a marker for BAM in the parenchyma (Lisi et al., 2017; Rajan et al., 2020; Santegoets et al., 2020). During the acute phase of stroke when peripheral myeloid cell infiltration was still mild and did not express CD163 in the rat model, transcriptome analysis of CD163^+^ BAMs identified an upregulation of HIF-1 pathway and an induction of genes encoding for extracellular matrix and leukocyte chemo-attractants. Furthermore, CD163^+^ BAMs are involved in recruiting granulocytes, promoting vascular endothelial growth factor (VEGF) expression, and inducing vascular leakage during the 24 h post-ischemia (Pedragosa et al., 2018). Another group further investigated the dynamics of BAMs after cerebral ischemia in rodents and humans, revealing a significant fivefold increase of CD163^+^ BAMs with inflammatory phenotype in the perivascular and meningeal spaces at 3 days post-ischemia (Rajan et al., 2020). Furthermore, CD163^+^ macrophages migrated from perivascular into ischemic parenchyma where they acquired pro-inflammatory phenotypes with the lack of Arg-1 and high inducible nitric oxide synthases (iNOS) expression. During the sub-acute phase of ischemia, a limited replacement of BAMs with peripheral monocytes which mature into CD169^+^ macrophages had also been determined in chimeric mice (Rajan et al., 2020). CD169^+^ macrophages have been revealed to act in concert with DCs to differentially affect adaptive immune responses to pathogens and self-antigens (Grabowska et al., 2018). Of note, it has been mentioned above that bone-marrow-derived precursors with the potential to enter CNS were not be able to spontaneously enter the blood stream unless isolated and transplanted artificially (Ajami et al., 2007), so studies using these techniques to establish BM chimeras had to be interpreted with caution.

### The Role of Peripheral Immunity in Ischemia/Hypoxia-Induced Neuroinflammation

#### Routes for Peripheral Leukocytes Infiltration

Hypoxia induced by high-altitude and disorders such as stoke, Alzheimer’s Disease (AD), cardiac arrest, and respiratory distress among many others, disrupts the integrity of BBB. Increased permeability of BBB was observed in mice after 6 h of 7% O_2_ hypoxia (Li et al., 2011). When exposed to 24 and 48 h of 8% O_2_, increased BBB leakage to sodium fluorescein was also demonstrated in mice (Schoch et al., 2002; Bauer et al., 2010). Notably, the opening of BBB has been found within the first hour of hypoxia in rat models (Witt et al., 2008). The molecular mechanisms of hypoxic barrier disruption have been studied intensively and HIFs, as the master regulators of hypoxic response, are suggested to play crucial roles. It has been revealed that in hypoxic models, HIF-1α accumulation induced BBB hyperpermeability and tight junction disorganization by activating its downstream targets VEGF and inducible nitric oxide synthesis 2 (iNOS2), resulting in a significant increase of FITC-dextran permeability and a decrease of ZO-1 expression in adult rat brain endothelial cell culture. Consistently, inhibition of hypoxia-induced HIF-1α accumulation by 3-(5′-hydroxymethyl-2′-furyl)-1-benzylindazole (YC-1) was demonstrated to be beneficial to protected BBB against hyperpermeability (Yeh et al., 2007). Moreover, HIF-1α has been revealed to induce chemo-attractant protein (MCP)-1 and MCP-5 expression in astrocytes, which might be involved in recruiting peripheral monocytes into the brain (Mojsilovic-Petrovic et al., 2007). In multiple neurodegenerative diseases (multiple sclerosis, AD, etc.) or cardiac arrest, trafficking of peripheral monocytes/macrophages, neutrophils, lymphocytes, or NK cells into the CNS through disrupted BBB took place to modulate cognition and disease progression (Larochelle et al., 2011; Corraliza, 2014; Zhang et al., 2018; Zhu et al., 2018).

In addition to the parenchymal vessels of BBB, the meningeal blood circulations and epithelial cells of CP provide alternative routes for peripheral infiltration, serving as educational gates for circulating or border-resident cells to invade the parenchyma in response to cytokines/chemokines released from injured brain or to skew immune cells toward certain phenotypes (Ransohoff et al., 2003; Shechter et al., 2013a). The molecular/cellular basis facilitating leukocytes infiltration through meninges and CP has been revealed in EAE during which significant hypoxia has been observed and related to neuroinflammation-induced functional deficits (Davies et al., 2013). The trafficking of effector T cells through the meninges in EAE was successfully recorded in a real-time manner by the intravital two-photon imaging. The infiltrated T cells were observed to get arrested and crawled on the luminal surfaces of leptomeningeal vessels followed by rolling along the inner surface of the vessels and pial membranes, from where the incoming T cells invaded the parenchyma to trigger neuroinflammation (Bartholomaus et al., 2009). The detachment and reattachment of effector T cells with leptomeninges has been reported to be determined by their contact with meningeal macrophages through integrin α4β1 (VLA-4) and lymphocyte function-associated antigen-1 (LFA-1) binding to their respective ligands on resident macrophages (Schlager et al., 2016). Moreover, ICAM-1 and vascular cell adhesion molecule-1 (VCAM-1) that constitutively expressed on epithelium but not endothelial cells in CP were up-regulated with *de novo* induction of mucosal address in cell adhesion molecule-1 (MAdCAM-1) expression of CP epithelial cells and thus mediated the binding of leukocytes via LFA-1 and alpha4-integrin during EAE (Steffen et al., 1996).

Multiple peripheral immune cell types, including DCs, T cells, monocytes/macrophages, granulocytes, and mast cells, have been reported to accumulate at the border of the brain or invade the parenchyma through the meningeal or choroidal vessels under ischemic/hypoxic conditions (Benakis et al., 2018). However, little is known about how the leukocytes are recruited into the brain through meninges and CP. In neonatal hypoxic ischemia (HI) injury, the expression of stromal cell-derived factor 1 (SDF-1 or CXCL12), a chemotaxic for CXCR4-expressing bone marrow-derived cells, was dramatically upregulated in CP and by mesothelial cells of pial meninges, which might be critical to the recruitment of circulating leukocytes into the brain (Miller et al., 2005). In stroke, the CCL2 gradient between CP and peri-infarct cortex, which was produced by parenchymal macrophages and microglia, contributed to the trafficking of infiltrated T cells from CP to peri-infarct cortex (Llovera et al., 2017).

In brief, under cerebral hypoxic conditions, various routes, including altered BBB, meninges, and CP, provide entries for the peripheral immune cells and molecules to invade the CNS to function.

#### Hypoxia Affects Peripheral Immune Cells Proliferation and Phenotype

Both physiological and pathological hypoxia have been described to function in regulating peripheral immunity and inflammation partially through their effect on physiological (bone marrow, lymphoid tissue, placenta, intestinal mucosa, etc.) and pathological (tumors and chronically inflamed, infected or ischemic tissues, etc.) immunological niches (Taylor and Colgan, 2017). A series of studies on peripheral immunity of temporary and native residents in Tien Shan and Pamir mountains showed that hypobaric hypoxia differentially affected the number, activity, or proliferation of T, B, NK, and phagocytic cells as well as peripheral cytokine profile (Mishra and Ganju, 2010). In a study on the alterations of immune system induced by acute and chronic exposure to high altitude, T lymphocytes were found significantly decreased during both acute and chronic hypoxia, which was totally attributed to CD4^+^ T cells reduction. Furthermore, an impairment of homeostatic Th1/Th2 balance occurred, evidenced by decreased expression of Th1 cytokine interferon-γ (IFN-γ) and Th1 marker CXCR3. On the contrary, the number of NK cells was increased, while their cytotoxic activity was not affected by high-altitude hypoxia (Facco et al., 2005). In people with acute mountain sickness (AMS), the level of proinflammatory cytokines (IL-1ß, IL-6, and TNF-α) was higher than that of the non-AMS group (Wang et al., 2018). Analysis of the transcriptome of the AMS and non-AMS group revealed that immune and inflammatory responses were overrepresented in AMS participants. Anti-inflammatory cytokine IL-10 was downregulated while inflammatory cytokines IF17F and CCL8 were upregulated in individuals of AMS. Consistently, the serum IL-10 concentration of AMS patients was also markedly decreased (Liu et al., 2017). Collectively, these data showed that high-altitude hypoxia induced peripheral inflammation by mobilizing multiple subsets of immune cells and enhancing circulating pro-inflammatory cytokines.

Importantly, HIFs have been demonstrated to be widely expressed in multiple immune cells as lymphocytes, macrophages, NK cells, and neutrophils, which supported an active role of HIFs in modulating peripheral immunity and inflammation. Altered activity of the pathways controlled by HIFs, in turn, affect the course of inflammation through the regulation of immune cell development and function (Taylor et al., 2016). When HIF1 or HIF2 was deleted in discrete immune cell subpopulations in mouse models, both innate and adaptive immunity were affected. The regulatory role of HIFs on immune cells is cell type-dependent, which influenced immune cell gene expression and downstream effector function (Cummins et al., 2016; Taylor et al., 2016; Taylor and Colgan, 2017). HIFs could also aid in immune cell adaptation to low oxygen by orchestrating a metabolic switch that allows cells to survive in hypoxia environment (Mcgettrick and O’Neill, 2020).

#### Peripheral Inflammation Participates in Neuroinflammation in Hypoxic Brain

Critical roles of the peripheral inflammation have been implied in AMS, especially in neuroinflammation. In a study of 283 hikers walking the Everest Base Camp Trek, some 87% of subjects experienced at least one symptom of infection (coryza, cough, sore throat, diarrhea) during the study period. Especially, infection was more prevalent among AMS individuals, and the number of infection symptoms was positively associated with AMS incidence and AMS score (Murdoch, 1995). Similar results were observed in rat models that were pre-treated with lipopolysaccharide (LPS) to induce systemic inflammation prior to short severe hypobaric hypoxia (7000 m), leading to cerebral edema through increased aquaporin-4 (AQP4) in the cortex and astrocytes and water permeability in the astrocytes by Toll-like receptor (TLR)-4 and corticotrophin-releasing hormone (CRH) signaling. In this study, systemic inflammation was suggested to facilitate the onset of hypoxic cerebral edema through interaction of astrocyte and microglia by ignition of TLR4 and CRH/CRHR1 signaling (Song et al., 2016). Consistently, our group has published a series of data to address the role of peripheral inflammation in augmenting hypoxia-induced cerebral edema (Zhou et al., 2017; Han et al., 2020). We found that preexisting systemic inflammation rapidly induced the onset of brain edema upon 6 h of acute hypobaric hypoxia (AHH) exposure by disrupting BBB integrity, activating microglia, and increasing water permeability via AQP4, which elicited impaired cognitive and motor function in mouse models, whereas AHH exposure without LPS-induced systemic inflammation could only induce a slight brain edema within 24 h (Zhou et al., 2017). We also reported that hypobaric hypoxia-induced cerebral inflammation occurred more easily in mice with dextran sulfate sodium (DSS)-induced colitis, which was demonstrated by increased level of Iba-1 in microglia and IL-1ß, IL-6, and TNF-α in the brain (Han et al., 2020).

In addition to function in hypobaric hypoxia conditions, peripheral inflammation has also been depicted to exacerbate HI-induced neuroinflammation. In a preterm HI model of rat-pup, low-dose (0.05 mg/kg) LPS pre-conditioned pups were demonstrated to be more sensitive to HI-induced white mater (WM) injury by selectively upregulating neuroinflammation and BBB leakage in WM (Wang et al., 2010). Significant hypoxia has also been revealed in the grey matter of cerebellum and cortex EAE mouse model of MS that is characterized by CNS inflammation (Johnson et al., 2016). In EAE model, inflammatory infiltrates was correlated with EAE progression. The appearance of infiltrating monocytes was involved in progression to the paralytic stage of EAE and inhibition of CCR2-dependent recruitment of monocytes prevented progression from mild to severe (Ajami et al., 2011). Recently, Yu’s group has reported that platelet-derived growth factor receptor beta (PDGFRß) cells in the brain, especially Col1a1 and Rgs5 subgroups, function as initial sensors and mediators of LPS or poly(I:C)-induced external inflammation by secreting chemokine CCL2 to rapidly upregulate excitatory synaptic transmission across multiple brain regions (Duan et al., 2018).

#### Crosstalk Between the Peripheral and Cerebral Immune

Brain-immune interactions have been studied in perinatal HI brain injuries, which is associated with varying degrees of neurological sequelae. In the neonatal brain, HI induced inflammation in the parenchyma, during which the neuroglial cells were activated by stressed or necrotic neurons and then released matrix metalloproteinases (MMPs), proinflammatory cytokines, and chemokines to facilitate peripheral immune cells to infiltrate into the brain to mediate secondary neuronal death (Wang et al., 2010; Algra et al., 2013). Accompanied with microglia activation, migration of peripheral inflammatory cells including both innate (monocytes/macrophages, neutrophils, DCs) and adaptive (T, B cells) immune cells and their release of cytokines and chemokines have been suggested to be the principal features of acute HI-induced neuroinflammation in the developing brain (Liu and Mccullough, 2013; Bhalala et al., 2014). In a hypoxia-induced neonatal brain injury model, which was induced by subjecting postnatal day 0 (P0d) rat pups to systemic hypoxia, the number of leukocytes and monocytes (CCR2^+^/CD45^+^/CD11b^+^) was increased in the blood. In the brain, systemic hypoxia also elevated the number of monocytes through interrupted BBB. Notably, the onset of peripheral inflammation occurred earlier than that of brain inflammation and intraperitoneally injection of minocycline to suppress circulatory inflammation even after the onset of hypoxia was beneficial to neuroprotection (Min et al., 2017).

The crosstalk between microglia and infiltrating peripheral immune cells has been implied in multiple hypoxia-associated cerebral brain injuries. In EAE mouse model, microglia and infiltrating inflammatory cells were implied to respond differently upon environmental stimuli and coordinate to regulate the course of disease. During the early stage of EAE, CD4^+^ T cells infiltration and resident microglia activation were involved in disease initiation. When EAE developed to a clinical score of 3 and 4, the number of microglia dropped markedly, while the number of infiltrating monocytes increased and coincided with functional impairment (Ajami et al., 2011). Consistently, in murine models of HI brain damage, CD11b^+^ myeloid cells were markedly elevated, among which monocytes-derived macrophages (MDM) that infiltrated in the parenchyma were increased in severely damaged brain, while residential microglia cells were decreased in both mild and severe brain injury and functioned in neuroprotection (Min et al., 2020). A dynamic analysis of neonatal HI in *lys*-EGFP-*ki* mouse model, in which myeloid cells generated through definitive but not primitive hematopoiesis expressed EGFP, revealed the marked expansion of CD11b^+^ cells in the immature CNS after HI consisted of a significant proportion of infiltrating peripheral monocytes and granulocytes, with a temporally biphasic pattern of two distinct infiltration peaks at day 1 and day 7 after HI. However, in this study, the level of resident monocytes was obtained to be low throughout the process (Smith et al., 2018). Furthermore, interferon regulatory factor 4 (IRF4) myeloid cell conditional knockout in neonatal HI models increased brain tissue loss and behavioral deficits by promoting microglia toward pro-inflammatory differentiation and enhancing peripheral monocytes and neutrophils infiltration (Al Mamun et al., 2019). The CNS-residential and infiltrated circulating immune cells in hypoxic brain altered to collaborate in the progression of hypoxia-related encephalopathy.

A series of studies on the interactions between microglia and infiltrated circulating leukocytes in AD and MS have been reported by Serge Rivest and his colleagues. In the lesions of MS and mouse models, recruited T cells and microglia are in proximity and interact with each other, including T cells activation by microglia via antigen presentation and microglia activation by T cells. Notably, the crosstalk between different subsets of microglia (M1 or M2) and T helper cells has been implied to result in different outcomes (Strachan-Whaley et al., 2014). In Alzheimer’s disease, the dynamic alterations of microglia and monocytes have been reported by Dr. Rivest’s group and other labs, with monocytes and microglia cooperating to play roles in removing amyloid beta and being potential therapeutic targets for AD (Naert and Rivest, 2011; Theriault et al., 2015; Fani Maleki and Rivest, 2019). Many studies from the group of Serge Rivest contribute to update our understanding of the crosstalk between the peripheral and CNS immunity in neurodegenerative disease.

#### Controversial Roles of the Peripheral Infiltrated Leukocytes in the Brain

Multiple types of peripheral immune cells, including lymphocytes, granulocytes, and monocytes, have been revealed to accumulate in a hypoxic brain, however, little is known about the functional relevance of different immune cell subtypes, leading to an obscure understanding about the effect of brain-infiltrated peripheral immune cells.

On one hand, peripheral immune cells infiltration into the brain was suggested to be detrimental. In neonatal HI pups that were established by permanent ligation of carotid artery followed by 2 h of hypoxia, T cells and NK cells were reduced in spleen yet increased in brain. Splenectomy to remove the largest pool of peripheral immune cells, as well as NK cells inactivation, significantly alleviated HI-induced brain injury (Fathali et al., 2013). Rapid and significant influx of neutrophils, monocytes, and T cells was observed in LPS-pretreated inflammation-sensitized HI (LPS/HI) brain injury in newborn mouse models. Prophylactic depletion of neutrophils by anti-Ly6G antibody before and immediately after LPS/HI insults specifically blocked cerebral infiltration of neutrophils without interfering with monocytes or lymphocytes influx, which markedly suppressed LPS/HI-induced proinflammatory cytokines, matrix metalloproteinase (MMP-9), and brain tissue loss (Yao and Kuan, 2020). Additionally, accumulation of neutrophils in adult brain after stroke has also been documented, supported by a similar observation in a mouse model of stroke (Perez-De-Puig et al., 2015). Depletion of neutrophils reduced the infarct size in a rat model of brain ischemic injury (Matsuo et al., 1994).

However, the roles of leukocytes influx into ischemic/hypoxic brain remains inconclusive, for in certain cases, inhibition of peripheral infiltration has no effect on the improvement of outcomes, or even worsen the situation. In a focal ischemic stroke rat model, depletion of neutrophils by anti-polymorphonuclear (PMN) treatment failed to reduce cerebral MMP-9 expression or infarct size (Harris et al., 2005). ICAM expressed on endothelial cells are critical for cerebral neutrophil infiltration under ischemic/hypoxic conditions. Though the monoclonal antibody for ICAM-1 (enlimomab) successfully improved neurological functions with reduced lesion size by reducing cerebral neutrophil influx in ischemic stroke models of rat and rabbit (Zhang et al., 1994; Bowes et al., 1995), it has no beneficial effect in ischemic stroke patients, instead, it induced significantly worse outcomes (Enlimomab Acute Stroke Trial, 2001), suggesting the roles of cerebral neutrophil infiltration might vary in different species upon ischemia/hypoxia. Natalizumab, a well-characterized humanized antibody against CD49d, efficiently mitigated leukocytes migration into the CNS, but failed to either reduce infarct volume or improve outcomes in ischemic stroke mouse model and clinical trials (Simats et al., 2016; Elkind et al., 2020). Moreover, siponimod (BAF132), a sphingosine-1-phosphate receptor modulator, has demonstrated beneficial effects on MS treatment by blocking leukocyte egress from lymphoid organs (Kappos et al., 2018), however, in experimental stroke, though a marked reduction of T cell accumulation in the CNS was detected, BAF132 has no effect on lesion size or neuroprotectivity (Vogelgesang et al., 2019), which indicated distinct roles of infiltrated leukocytes in different types of hypoxic brain injuries.

The inconclusive effects of infiltrated circulating cells on hypoxic brain have been suggested to be determined by interactions among different immune cell types, with certain cell types playing beneficial roles. In HI neonatal mouse models, in contrast to the original hypothesis that reducing peripheral lymphocytes infiltration into the injured brain would promote neuroprotection, FTY720 or anti-CD3 treatment-induced decrease of T cells and particularly Foxp3^+^ regulatory T cells (Treg) infiltration coincided with increased brain infiltration of neutrophils and inflammatory macrophages and exacerbated HI-induced brain injury (Herz et al., 2018). Very recently, the beneficial effect of Treg on stoke recovery has been illustrated. Though spanning the acute, subacute, and chronic stages of ischemic stroke in mouse model, Treg significantly escalated from 5 to 35 days, a period during which functional recovery took play. Selective depletion of Treg in *Foxp3* ^*DTR*^ transgenic mouse by diphtheria toxin injection diminished oligodendrogenesis, white matter repair, and functional recovery after stroke. While boosting Treg cell number by administration of IL-2:IL-2 antibody complexes markedly improved white matter integrity and long-term stroke outcomes (Shi et al., 2021). Mechanistically, the crosstalk between Treg-derived osteopontin and integrin receptor on microglia enhanced microglial reparative capability, which promoted oligodendrogenesis and white matter integrity (Shi et al., 2021).

The complicated roles of peripheral infiltrating cells in ischemic/hypoxic brain were also manifested by their sex-specific manner. Inflammatory responses and injury outcomes in experimental neonatal HI models have been reported to diverge according to gender, with increased inflammation and monocyte infiltration in males compared to females (Hill and Fitch, 2012; Mirza et al., 2015). Interestingly, Gr-1 antibody administration reduced myeloid infiltration in neonatal HI brain and function in neuroprotection but had no effect in female subjects (Smith et al., 2018).

## Conclusion

Peripheral immune plays critical roles in both steady-state brain and cerebral disorders induced by hypoxia. Increasing evidence has been unravelling the complicated effects of peripheral immune in the development and progression of hypoxia-associated brain injuries and multiple brain-infiltrated cell types have been discussed. However, controversial results indicate that, instead of one single cell type dominating during the whole process, multiple cell types from the peripheral and the brain collaborated, with different populations functioning in different stages, to determine the outcome of hypoxia-associated brain injuries. Therefore, it is urgent to uncover the landscape of the infiltrated populations throughout the whole hypoxia process (acute, subacute, chronic) and explore the interactions among the infiltrated and CNS-residential cells, which will benefit the identification of stage-specific critical regulators and shed new light on the understanding of molecular mechanisms of hypoxia-associated brain injuries.
