# Supplementary material for: Multiple Roles of Peripheral Immune System in Modulating Ischemia/Hypoxia-Induced Neuroinflammation
Source: Front Mol Biosci. 2021 Nov 22;8:752465. doi: 10.3389/fmolb.2021.752465 (PMC8645603; doi:10.3389/fmolb.2021.752465)
Supplement: Supplementary file 1 [file Table1.pdf]

Responses of CNS-residential immune cells and peripheral inflammation to cerebral hypoxia under various pathological conditions

	**CNS-resident immune**<br>（Microglia & BAM）	**Peripheral immune**<br>(Peripheral inflammation & Infiltrated leukocytes)
**Hypoxia/ hypobaric hypoxia**	• Reactive microgliosis in acute and chronic hypobaric hypoxia *(Dheer et al., 2018; Shi et al., 2017);*<br>• Transition to M1 phenotype with increased expression of M1 markers and decreased level of M2 markers *(Butturini et al., 2019; Habib et al., 2013; Habib et al., 2014; Zhang et al., 2017)*<br>• Critical roles of microglia in maintaining BBB integrity under chronic mild hypoxia *(Sebok et al., 2019; Halder and Milner, 2019; Halder and Milner, 2020)*	• Infection was more prevalent among AMS individuals and systemic inflammation facilitated the onset of hypoxic cerebral edema *(Murdoch, 1995; Song et al., 2016; Han et al., 2020; Zhou et al., 2017; Zhou et al., 2017)*<br>• Increased permeability of BBB and induction of chemokines (MCP-1, MCP-5, *etc.*) in the brain under hypoxia and high-altitude conditions, which might be involved in peripheral infiltration *(Li et al., 2011; Bauer et al., 2010; Schoch et al., 2002; Witt et al., 2008; Mojsilovic-Petrovic et al., 2007)*
**Ischemia/ hypoxic ischemia (HI)/ ischemic stroke**	• Reactive microgliosis *(Ladeby et al., 2005; Yrjanheikki et al., 1998)*<br>• Dynamic transition to M1 phenotype *(Hu et al., 2012)*<br>• Microglia autophagy in mouse model of ischemic stroke *(Yang et al., 2015).*<br>• Dual roles of microglia in BBB disruption *(Adams et al., 2007; Jolivel et al., 2015; Kang et al., 2020)*<br>• An increase of BAM with proinflammatory phenotype in perivascular and meningeal space in ischemia, recruiting granulocytes *(Pedragosa et al., 2018; Rajan et al., 2020)*	• LPS-induced peripheral inflammation sensitized rat-pups to brain injury in preterm HI model *(Wang et al., 2010)*<br>• Migration of peripheral innate (monocytes/macrophages, neutrophils, DCs) and adaptive (T, B cells) immune cells and their release of cytokines and chemokines are the principal features of acute HI-induced neuroinflammation *(Nazmi et al., 2018; Bhalala et al., 2014; Liu and McCullough, 2013）*<br>• Accumulation of neutrophils in adult brain after stroke and mouse model *(Perez-de-Puig et al., 2015)*
**Multiple sclerosis (MS/EAE)**	• Reactive microgliosis *(Hemmer et al., 2002)*<br>• Dynamic M1/M2 transition in disease progression *(Chu et al.,2018; Brooke et al.,2020)*<br>• Pathogenic role in BBB disruption *(Adams et al., 2007)*<br>• Both microglia and BAM can encounter the infiltrated peripheral T cells to exacerbate MS injury *(Dong and Yong, 2019)*	• Inflammatory infiltrates was correlated with EAE progression *（Ajami et al., 2011）*<br>• A sustained infiltration of lymphocytes (T cells, neutrophils) and monocytes in EAE *(Larochelle et al., 2011; Schmitt et al., 2012)*
**Amyotrophic lateral sclerosis (ALS)**	• The large increase in microglia was attributed to CNS-resident microglia self-renewal *(Ajami et al., 2007)*<br>• M1/M2 microglia polarization determines ALS progression *(Geloso et al., 2017)*	• Systemic pro-inflammation is existed in ALS and associated with disease severity *(Beers et al., 2020; Keizman et al., 2009)*<br>• T lymphocytes and monocytes/macrophages infiltration into the spinal cord of ALS patients and mouse model *(Beers et al., 2011; Shiraishi et al., 2021; Zhao et al., 2013)*
**Alzheimer's Disease (AD)**	• Reactive microgliosis (Benveniste et al., 2001; Hansen et al., 2018; Zhang et al., 2021);<br>• Excessive activation of M1 microglia and dysfunction of M2 phenotype promotes AD progression *(Tang et al., 2016; Yao et al., 2019)*<br>• Microglia and BAM maintain an embryonic origin during AD *(Wu et al., 2021)*	• Both acute and chronic systemic inflammation is associated with an increase in cognitive decline in AD *(Holmes et al., 2009; Kamer et al., 2008);*<br>• Infiltration of T cells, monocytes, natural killer cells and neutrophils in AD patients and mouse models *(Bettcher et al., 2021; Cao and Zheng, 2018; Unger et al., 2018)*
